# Resistance to EGFR Inhibitors in NSCLC: Mechanistic Insights and Emerging Therapies

**DOI:** 10.3390/ijms27125197

**Published:** 2026-06-09

**Authors:** Rita Khoury, Chris Raffoul, Colette Hanna, Khalil Saleh, Annoir Shayya, Meriana Nahouli, Hady Ghanem

**Affiliations:** 1 Division of Hematology & Oncology, Lebanese American University Medical Center-Rizk Hospital, Beirut P.O. Box 11-3288, Lebanon; colette.hanna@lau.edu.lb (C.H.); anwar.shayya@lau.edu.lb (A.S.); miryana-nh@hotmail.com (M.N.);; 2 Department of Internal Medicine at VCU Health, Richmond, VA 23298, USA; chris.raffoul@vcuhealth.org; 3 International Department, Gustave Roussy Cancer Campus, 94800 Villejuif, France; khalil_saleh@live.com

**Keywords:** NSCLC, EGFR mutation, EGFR inhibitors resistance

## Abstract

Non-small cell lung cancer (NSCLC) accounts for up to 85% of lung cancer cases, with activating EGFR mutations present in 10–15% of Western and up to 35% of Asian patients. EGFR tyrosine kinase inhibitors (TKIs) have transformed management, with first-line osimertinib demonstrating a median progression-free survival (PFS) of 18.9 months and overall survival (OS) of 38.6 months in the FLAURA trial. However, resistance inevitably develops, most commonly via T790M mutations (~50% of cases after first- and second-generation TKIs), and after osimertinib, through diverse mechanisms including C797S mutations, MET/HER2 amplification, and histologic transformation. Emerging strategies to overcome resistance include next-generation TKIs, combination targeted therapies, downstream pathway inhibitors, immunotherapy approaches, and antibody–drug conjugates. Understanding these mechanisms is critical for optimizing patient outcomes and guiding personalized therapeutic approaches. This review discusses current strategies to delay or overcome resistance and highlights emerging therapeutic avenues with the potential to reshape the management of EGFR-mutant NSCLC.

## 1. Introduction

Lung cancer represents around 12% of new cancer diagnoses in the United States and is estimated to account for 124,730 deaths by the end of 2025, making it the leading cause of cancer mortality both in the US and worldwide [1]. Non-small cell lung cancer (NSCLC) comprises approximately 85% of all lung cancer cases [2]. Molecular profiling has significantly advanced the management of NSCLC by identifying key driver mutations and rearrangements, enabling the development of personalized biomarker-driven treatment approaches. Among these, epidermal growth factor receptor (EGFR) mutations were among the earliest actionable alterations discovered in NSCLC.

Tyrosine kinase inhibitors (TKIs) targeting EGFR have demonstrated substantial clinical benefit, with successive generations showing improved efficacy and safety. Nevertheless, despite the remarkable responses observed, the majority of patients ultimately develop acquired resistance. While several mechanisms of resistance have been elucidated, the underlying cause remains unclear in approximately 30% of cases, underscoring the complexity of tumor biology. International guidelines—including those from the American Society of Clinical Oncology (ASCO), the National Comprehensive Cancer Network (NCCN), and the European Society for Medical Oncology (ESMO)—recommend upfront treatment with osimertinib. However, the primary challenge lies in resistance patterns related to EGFR mutations, making a thorough understanding of these mechanisms crucial.

## 2. EGFR

EGFR, also known as ErbB1/HER1 is a member of the EGFR family, which includes ErbB2/HER2/Neu, ErbB3/HER3, and ErbB4/HER4 [3].

This proto-oncogene was actually discovered by accident during an experiment on Nerve growth factor (NGF). Stanley Cohen and Rita Levi-Montalchini were observing the effect of NGF on newborn mice when they noticed teeth appearance and precocious eyelid opening in the mice treated with the NGF fractions [4]. They focused on the unexpected side effects to try and isolate the sole component. This phenomenon was found to be due to the rapid stimulation of epidermal growth as well as keratinization and thus the name behind the epidermal growth factor [5]. Soon afterwards, Cohen realized that the discovered murine EGF can also boost DNA synthesis fibroblasts from humans; therefore, the human EGF was discovered [4]. Subsequently the question arose around its mechanism of action. Initially, receptors on the plasma membrane were thought to bind peptide hormones that are then released into the extracellular environment. Nevertheless, Cohen demonstrated that EGF in fact binds a membrane-bound protein, EGFR, and then the pair is internalized and degraded [6,7,8].

It is well-recognized that the ErbB family stimulates several pro-oncogenic biological routes, comprising angiogenesis, cell proliferation, inhibition of apoptosis, cell motility, and metastasis.

It is important to mention that the link of EGFR to cancer was first documented when the EGFR peptides were found to closely match the vErb-B oncogene of the avian erythroblastosis virus (AEV), associating EGFR mutations with cancer [9,10]. Moreover, not only EGFR aberrations but also overexpression were linked to cancer progression, first in carcinoma [11,12] and later on in malignant gliomas [13], NSCLC [14], and sarcomas [15]. EGFR levels also predict the tumor grade, possible relapse and patient prognosis [16,17].

Notably, EGFR mutations are more frequently observed in females compared to males and in individuals who have never smoked versus smokers—highlighting important demographic and environmental factors in mutation prevalence and disease risk [3].

a. EGFR expression:

The EGFR gene lies on chromosome 7 short arm q22, and it spans 110 Kb of DNA. It has a total of 28 exons [18,19]. Each normal cell approximately contains 40,000 to 100,000 binding sites for EGF [20]. The EGF receptor is in fact regulated by EGF itself. It stimulates the expression of ETF (EGFR- specific transcription factor) to increase EGFR RNA expression and transcription by 5–10 fold [21,22]. Other proteins also modulate the EGFR promoter, and they include E1A [23], AP2 [24], and Sp1 [25].

b. EGFR structure:

The EGFR precursor is initially synthesized as a single-chain polypeptide with a length of approximately 1210 residues [10]. During post-translational processing, a proteolytic cleavage event occurs near the N-terminal region of the precursor, resulting in the removal of a portion of the protein. This cleavage generates the mature form of EGFR, which consists of 1186 residues [26].

The extracellular region is composed of 621 amino acids and is further subdivided into four distinct domains, each with its own specific functions and characteristics, the most important of which is the intracellular domain comprised of 542 amino acids and contains three components [27,28]: a flexible juxtamembrane segment (~40 aa) that connects the TM to the tyrosine kinase domain and regulates EGFR activation and signaling. The tyrosine kinase domain (exons 18–24) plays a crucial role in EGFR signaling by phosphorylating tyrosine residues in both the EGFR itself and downstream signaling proteins. The tyrosine kinase domain can be further divided into two lobes: the N-lobe, which is mainly composed of beta-sheet structures, and the C-lobe, which consists of mainly alpha-helical structures. The ATP-binding site is situated between these two lobes, enabling ATP binding and subsequent phosphorylation events [29]. Transautophosphorylation happens upon the interaction of the N-lobe of one receptor to the C-lobe of the other [30].

The C-Terminal Tail (exons 25–28) facilitates signal transduction and regulates cellular processes involved in cell growth, survival, and proliferation [28].

c. Physiological Role of EGFR

EGF receptors have been recognized in almost all cell types except the hematopoietic cells. These receptors are found in abundance in the thyroid, lung, liver, skin, brain, placenta, and fetal membranes [31]. The EGFR family is vital to the normal embryogenesis process [32]. It plays an important role in rat corneal development [33], skin maturation, hair cycling, and hair follicle development [34]. In addition, the EGFR gene is found to play a major role in mammary ductal development. In fact, in female mice with impaired tyrosine kinase activity, defective ductal growth was observed and failure to develop proper mammary glands, leading to malnutrition and pups’ death [35,36,37].

Moreover, and although the EGF mechanism of action on CNS neurons remains unclear, evidence on their effects is accumulating [38]. Receptors are found in high levels in the developing astrocytes but disappear in fully mature astrocytes [39,40]; however, and interestingly, EGFR is re-upregulated in the case of tumor development, ischemia, or neurodegenerative diseases [41].

d. EGFR mutations:

EGFR mutations are found in numerous tumor types; however, the alterations are not quite similar among them. They vary across tumor types, and they are not evenly dispersed along the gene, leading to the hypothesis of “hot spots” [3] and the fact that certain tumor types favor certain mutations [3,42]. For instance, glioblastomas exhibit mutations in the ectodomain region (exons 2–7), whereas NSCLC predominantly harbor these aberrations in the kinase domain. Colorectal less frequently harbor EGFR mutations but rather gene amplification in around 50% of the cases [43,44]. EGFR overexpression is also found in 40–80% of NSCLC [45]. Mutations are rarely seen in the transmembrane domain.

In the ectodomain, EGFR mutations often result in the loss of the inhibitory regulatory domains for dimerization; the viral homolog v-ERBB is the most known EGFR mutation in the ectodomain.

In parallel, the most frequently seen EGFR mutation in the kinase domain is L858R, and it makes up around 40–50% of all mutations in the tyrosine kinase domain [46,47]. L858R is considered a “classical” activating mutation that occurs specifically in exon 21. It results in the substitution of leucine (L) with arginine as position 858 within the activation loop of the tyrosine kinase domain, and it demonstrates 50-fold higher kinase activity, as well as increased Km value for ATP compared to wildtype EGFR. These combined effects lead to uncontrolled cell proliferation, survival, and cancer development [48,49]. Another classical activating mutation, that is usually seen in NSCLC, is the exon 19 in-frame deletion [50].

**Figure 1:**
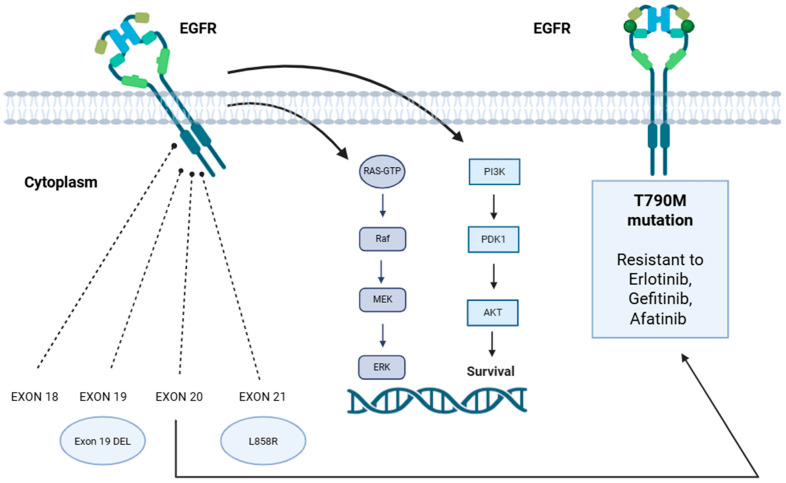
Illustration of EGFR signaling pathway, including common EGFR mutations and downstream signaling. The green section in the figure highlights the areas where ligands bind and mutations typically occur, whereas the teal sections represent the transmembrane and intracellular domains triggering downstream signaling.

T790M, known as the “gatekeeper residue” mutation (which will be discussed later in this paper), has been recently observed in patients who developed resistance to TKI after initial response. The T790M mutation involves a substitution of threonine (T) with methionine (M) at position 790 within exon 20 of the EGFR gene (Figure 1). This mutation is of particular significance because it not only increases EGFR phosphorylation levels but also imparts resistance to TKIs, such as gefitinib and erlotinib, which are commonly used as targeted therapies for EGFR-mutant NSCLC [47,51,52]. The exact mechanism by which the T790M mutation confers resistance to TKIs is not fully understood. One proposed explanation is that the bulkier side chain of methionine at position 790 introduces steric hindrance, preventing the binding of TKIs to the EGFR kinase domain. This steric hindrance disrupts the binding pocket and reduces the affinity of TKIs for the mutant EGFR, rendering them less effective in inhibiting the aberrant signaling [53].

## 3. Clinical Trials

In Caucasian patients, approximately 10–15% have been found to harbor EGFR mutations, while in Asian patients, the prevalence is higher, ranging from 30–35%. These mutations are more commonly observed in patients who have never smoked or have a history of light smoking. Deletions in exon 19 account for approximately 40–50% of EGFR mutations, while L858R substitutions in exon 21 carry around the same number of EGFR mutations [54]. The detection of these mutations and later on the development of EGFR-tyrosine kinase inhibitors (EGFR-TKIs) have radically changed the treatment landscape of EGFR-mutated NSCLC and eventually improved its prognosis.

First, gefitinib is a first-generation reversible oral EGFR-TKI. Efficacy was reported in the IPASS trial, in which 1217 patients with advanced pulmonary adenocarcinoma who never or lightly smoked, were assigned to receive gefitinib or carboplatin + taxol. The PFS at 1 year improved from 6.7% to 24.9% in the ITT; however, subgroup analysis showed that EGFR-negative patients had a significantly shorter PFS with gefitinib [55].

Afterwards, two phase III trials exclusively enrolled EGFR-mutated NSCLC. WJTOG3405 is an open label, randomized, and phase III study that enrolled patients with advanced NSCLC, with either exon 19 deletion or L858R point mutation, to receive gefitinib or cisplatin plus docetaxel. A longer PFS was observed in the gefitinib arm. The most frequent adverse events consisted of diarrhea, skin toxicity, and liver dysfunction [56].

Another phase III study, reported by Maemondo et al., compared gefitinib to standard chemotherapy (carboplatin + taxol) in the first line setting in patients with non-T790M EGFR-mutated NSCLC. The median PFS doubled from 5 to 10 months in the gefitinib arm with significantly higher 1-year and 2-year rates of PFS. Similarly, the ORR increased from 30 to 73% [57].

Second, erlotinib, another first-generation TKI showed clinical benefit in EGFR + NSCLC. In the OPTIMAL trial, 154 patients from China were randomly assigned to receive erlotinib or gemcitabine plus carboplatin [58]. The erlotinib arm exhibited improved PFS with 13.1 months vs. 4.6 months in the chemotherapy group. The ORR improved, but the OS data were not significantly different; however, patients who received post-study treatment or received TKI along the course of their treatment had better OS compared to those who did not [59]. In addition, the ENSURE trial showed a doubling of PFS and ORR in Asian patients with EGFR mutation-positive NSCLC treated with erlotinib [60]. Another phase III trial, EURTAC, showed an improved PFS (9.7 vs. 5.2 mo), higher response rate (64% vs. 18%), and fewer adverse events with erlotinib compared to chemotherapy (cisplatin plus docetaxel) in the non-Asian population [61].

For the second-generation TKIs, afatinib, an irreversible TKI, has been shown to have a clinical benefit in different trials. The LUX-Lung 3 trial compared afatinib to cisplatin plus pemetrexed in advanced lung adenocarcinoma patients. The PFS significantly increased (11.1 mo vs. 6.9 mo) with an ORR of 56% vs. 23%. Adverse events were consistent with those previously seen with other TKIs. Better symptomatic control as well as improved quality of life were also observed [62,63]. Comparable results of superior PFS and ORR were also reported in LUX-lung6 [64].

Dacomitinib is another second-generation TKI approved for the frontline treatment for NSCLC with EGFR mutation. Although it showed superior PFS (14.7 vs. 9.2 mo) and improved OS (34 vs. 27 mo) when compared to gefitinib, more grade 3/4 dermatitis and diarrhea were observed with its use [65,66].

Osimertinib, a third-generation EGFR-TKI, has proven highly effective in patients with EGFR-mutant NSCLC who harbor the T790M resistance mutation. In the pivotal phase III AURA3 trial, osimertinib achieved a median progression-free survival (PFS) of 10.1 months, significantly longer than the 4.4 months observed with platinum-pemetrexed chemotherapy (hazard ratio [HR] 0.30; *p* < 0.001). In addition, among patients with central nervous system (CNS) metastases, the PFS was extended from 4.2 to 8.5 months (HR 0.32). Osimertinib also demonstrated a more favorable safety profile, with grade ≥ 3 adverse events occurring in 23% versus 47% with chemotherapy [67].

Beyond the T790M-positive setting, the FLAURA trial established osimertinib as superior to first-generation TKIs in treatment-naïve patients with activating EGFR mutations, showing significantly longer PFS [68].

## 4. Mechanisms of Resistance to TKIs

Despite advances, almost all patients will eventually develop resistance to TKIs. Resistance mechanisms can be diverse and complex, and individual patients may exhibit different mechanisms or a combination of mechanisms. Understanding these mechanisms is crucial for developing effective strategies to overcome or prevent resistance and improve treatment outcomes for NSCLC patients.

### 4.1. Primary Resistance

While exon 19 deletion and L858R represent the most common EGFR mutations and confer sensitivity to TKIs, other primary point mutations, although less common, do not show any sensitivity. Intrinsic resistance to TKI is usually the result of the presence of a non-sensitive mutation. Exon 20 insertion accounts for 1–10% of EGFR mutations, and although phenotypically similar, NSCLC patients harboring exon 20 mutations do not respond to first-generation TKIs, with a reported response rate barely reaching 5% [69,70].

It is worth mentioning the recent accelerated approval of sunvozertinib in locally advanced NSCLC with EGFR exon 20 insertion mutations in adult patients whose disease has progressed on or after platinum-based chemotherapy. Oral irreversible TKI has potent activity against EGFR mutations. This approval was based on the WU-KONG1B trial, which was a multinational, open-label, and dose randomized phase II trial showing an objective response rate of 46% and a median duration of response of 11.1 months [71].

Amivantamab is a bispecific antibody targeting both EGFR and MET receptors. It showed PFS benefit in the first line setting when added to chemotherapy in patients with EGFR exon 20 insertion [72], as well as durable response rates later line options after chemotherapy failure [73]. Similarly, mobocertinib showed benefit in subsequent therapies in patients with exon 20 insertion mutations, gained accelerated approval in 2021, but failed to satisfy confirmatory requirements by the FDA and was formally withdrawn by end of 2024 [74].

Most recently, zipalertinib, is under investigation, as a phase I/IIa study demonstrated its activity in heavily pretreated NSCLC patients harboring EGFR exon 20 insertions [75].

Another EGFR mutation in the extracellular domain is the EGFRvIII. It leads to structural changes affecting the intracellular domain and the ATP pocket and eventually results in TKI resistance [76].

Primary resistance can also be the result of concurrent molecular or genetic modifications that decrease the sensitivity to TKIs. Patients who present a spectrum of responses despite identical mutations lead to the theory that different responses may be due to different apoptotic machinery. One example is BIM or BCL2L11, which is a proapoptotic Bcl-2 family member. Different levels of BIM are due to a genetic polymorphism that leads to altered function. Patients who harbor this polymorphism show inferior response compared wildtype tumors. Nakawaga et al. demonstrated that the histone deacetylase (HDAC) inhibitor vorinostat helps sensitize resistant cells that harbor BIM polymorphisms. Therefore, when combined with EGFR-TKI, it restores BIM function and tumor sensitivity to TKI [77].

In addition, the EGFR-mutant NSCLC with lower BIM expression displayed a shorter PFS and decreased response [78,79,80]. In the EURTAC trial, tumors with higher BIM expression had a longer PFS and OS, making BIM not only a mediator of sensitivity but also a good biomarker [81].

Another example of intrinsic resistance is illustrated by increased basal levels of CRIPTO1, knowns as TDGF1 (teratocarcinoma-derived growth factor), a cell-membrane-attached protein that, when present, is able to diminish the sensitivity to TKIs and consequently promote epithelial-to-mesenchymal transition and eventually stimulate AKT and MEK signaling [82].

### 4.2. Acquired Resistance to EGFR-TKIs in NSCLC

**Figure 2:**
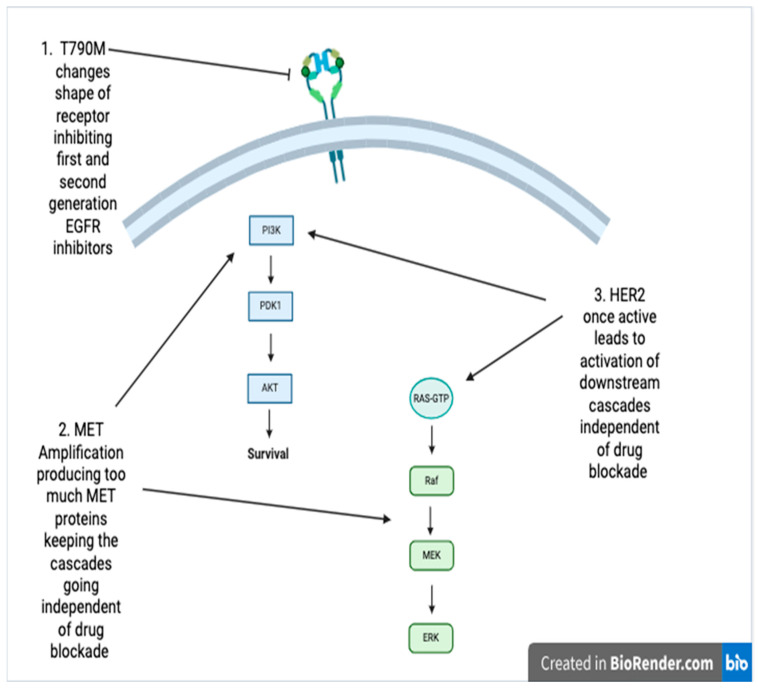
Illustration showing the common mechanisms leading to EGFR resistance.

**Figure 3:**
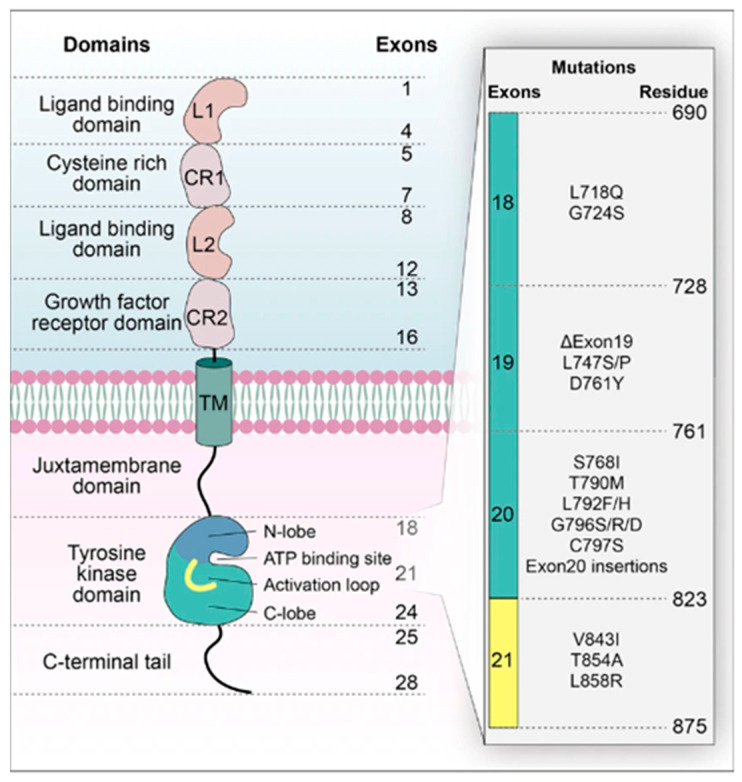
Brief description of EGFR domains and the molecular mechanisms of acquired resistance. The intracellular domain contains a juxtamembrane domain, tyrosine kinase domain, and multiple C-terminal tyrosine residues. Multiple mutations within the tyrosine kinase domain are associated with resistance and sensitivity to EGFR-TKIs. Source: adapted from Kunyu Shi, 2022 [83], “Emerging Strategies to Overcome Resistance to third-generation EGFR inhibitors”, Springer Nature Link.

Acquired or secondary resistance is usually the result of prolonged exposure to TKIs. Numerous mechanisms have been suggested to contribute to the drug resistance including secondary mutations in the EGFR gene, activation of alternative pathways, or tumor phenotypic transformation as seen in Figure 2 and Figure 3.

a. Secondary mutations

The T790M in exon 20 is the most commonly acquired mutation, observed in almost 50% of the biopsies obtained in patients known to have exon 19 or L858R mutations following disease progression on first-generation TKIs [84,85,86].

In addition to the previously mentioned substitutions, it alters the affinity of EGFR to ATP rendering it the favored substrate as compared to the competitive TKI [52,87].

Even though the emergence of the T790M mutation is known and proven post TKI therapy, not much is known concerning how resistant clones actually evolve. Are there pre-existing drug-resistant EGFR-T790M-positive clones, or do cells evolve genetically and acquire de novo resistant mutations after a period of drug sensitivity?

Hata et al. tried to answer this question by studying different clones and monitoring their response to treatment with TKIs. They treated different cells with similar doses of gefitinib and monitored the response and interval time to emerging resistant patterns. The variable time to resistance ranged from 2–3 weeks to more than 24 weeks, from early-resistant clones to intermediate and late-emerging EGFR T790M clones leading to the conclusion that both pre-existing and de novo EGFR-T790M mutations co-exist [88].

It is important to mention that in preclinical models, positive results suggest that combining afatinib (second-generation TKI) with cetuximab (anti-EGFR antibody) could be a promising therapeutic approach in overcoming T790M-mediated resistance [89]. Nevertheless, these data need to be validated in clinical trials before becoming standard practice.

Another way of overcoming this secondary mutation is the use of third-generation TKIs. As mentioned previously, osimertinib, also known as AZD9291, is a third-generation TKI that showed benefit in previously treated NSCLC patients who developed the T790M mutation [68]. Given that osimertinib is a key player in such a setting, resistance to this drug has been explored in attempt to overcome it and regain clinical benefit. EGFR C797S is the most common pathway to resistance; it is a tertiary mutation that affects the binding site of the osimertinib, rendering all third-generation inhibitors much less potent [90].

The second most common pathway of resistance is the insurgence of MET amplification, which has been reported following treatment with first-generation TKIs. It accounts for about 20% of acquired resistance causes. Engelman et al. reported the emergence of MET proto-oncogene amplification in 4 out of 18 NSCLC patients who developed resistance to gefitinib or erlotinib [91].

MET receptor overexpression maintains ERBB3 phosphorylation and results in the activation of downstream signaling pathways, triggering the PI3K/Akt signaling and thus promoting cell proliferation, growth, and survival. Of note, this is an EGFR-independent process, as uncontrolled MET activation can be achieved regardless of EGFR inhibition/blockade status, facilitating the hostile behavior of EGFR–TKI resistant clones [92].

Likewise, and though infrequent, ErbB2 gene mutations are also detected and identified as a new mechanism of resistance that is responsible for almost 2% of NSCLC-resistant cases independent of T790M; these HER-2 mechanisms are generally observed after the use of third-generation TKIs. ErbB2 activation is strongly dependent on EGFR transphosphorylation; however, when mutated, it becomes self-regulating and induces resistance [93].

Moreover, Insulin-like growth factor 1 receptor (IGF-1R) is thought to be involved and has been actually associated with resistance to EGFR-TKI by possibly conveying extracellular survival signals to downstream receptors such as MAK and AKT. Choi et al. tested the effect of co-inhibiting IGFR as well as EGFR. This simultaneous inhibition induces apoptosis and enhances gefitinib’s effects, suggesting a potential overcoming approach [94].

b. Phenotypic transformation

Besides secondary mutations and alternative pathway activation, a third mechanism of resistance has been actually described. Sequist LV et al. actually performed histologic analyses from 37 patients with TKI-resistant NSCLC in order to elucidate mechanisms of acquired resistance. Surprisingly, 5 out of 37 patients changed into small cell lung histology (SCLC) and were actually sensitive to standard SCLC treatment. There is no exact mechanism that explains the underlying phenomenon; however, researchers suggest a transformation of pre-existing cells rather than de novo clones’ development, as patients retained their original EGFR mutations [95]. In recent years, multiple studies have corroborated the occurrence of small cell lung cancer transformation as a relatively uncommon but clinically significant mechanism of resistance in EGFR-mutant non-small cell lung cancer. Recent series reported such occurrence to be roughly around 3–10% of cases. For example, Saalfeld et al., 2024 [96] included a multicenter retrospective analysis of 47 patients discussing outcomes and treatment responses, documenting the transformation in 7% of the cases.

Additionally, epithelial–mesenchymal transition (EMT), a process in which cells undergo a biological shift from tightly connected immotile cells to a more loosely adherent form with greater motility, promoting invasive properties, is greatly related to phenotypical transformation [97]. Loss of E-cadherin expression is a hallmark of EMT. In the TRIBUTE trial, among patients who received chemotherapy plus erlotinib, the time to progression was longer in patients with retained E-cadherin staining, supporting the preclinical hypothesis that the EMT plays a major role in the context of resistance [98]. Along the same lines, in the EMT setting, AXL upregulation and Hedgehog (Hh) pathway activation were also implicated in tumor progression and acquired EGFR–TKI resistance [99,100].

## 5. Strategies to Overcome EGFR-TKI Resistance in NSCLC

The identification of resistance mechanisms to EGFR-TKIs has led to the development of therapeutic strategies aiming to restore sensitivity and bypass the resistant pathways. These approaches target specific molecular alterations, use combination therapies, and explore new treatment modalities.

### 5.1. Fourth-Generation TKIs: Overcoming C797S

As resistance to osimertinib frequently arises through tertiary mutations such as C797S, a new wave of fourth-generation EGFR inhibitors is in development to address this challenge.

Preclinical studies highlight BLU-945 and BLU-701 as promising agents. BLU-945 has demonstrated potent wildtype sparing activity against both double (e.g., L858R/T790M) and triple (L858R/T790M/C797S) EGFR mutations, with high in vivo antitumor efficacy, including intracranial activity in osimertinib-resistant models [101,102]. BLU-701, while primarily active against EGFR mutations with C797S, lacks some of the broader mutational coverage of BLU-945 but still induces significant tumor regression. Importantly, combinations, such as BLU-945 plus BLU-701, have demonstrated prolonged and enhanced tumor regression in osimertinib-resistant triple-mutant xenograft models [101].

Additional fourth-generation inhibitors currently under study include JIN-A02 and QLH11811. JIN-A02 has shown preclinical activity against EGFR triple mutants, including C797S, with promising potency and selectivity, while QLH11811 is being investigated for its ability to target osimertinib-resistant EGFR mutants in both in vitro and in vivo models [103,104].

Both BLU compounds entered early-phase clinical testing; however, the Phase 1/2 SYMPHONY trial of BLU-945 was terminated due to sponsor-related reasons, while the HARMONY trial of BLU-701 was discontinued because of a lack of efficacy [105,106].

c. Allosteric Inhibitors: EAI045

Allosteric EGFR inhibitors, such as EAI045, represent a different strategy. Instead of competing at the ATP-binding catalytic site, they bind an allosteric pocket outside this region, thereby potentially avoiding steric hindrance caused by mutations like C797S, which impair the binding of ATP-competitive inhibitors [107].

EAI045 was the first allosteric inhibitor reported to selectively target mutant EGFR, showing synergistic activity in preclinical models when combined with cetuximab. Another compound, JBJ-04-125-02, was subsequently developed to improve on the pharmacologic limitations of EAI045 and demonstrated greater potency, including activity against EGFR L858R/T790M/C797S triple mutants in preclinical studies [107]. Despite these promising data, both agents remain in the preclinical stage, with no clinical translation to date.

Collectively, these advances illustrate the evolution of EGFR-targeted therapy across successive generations of inhibitors, each designed to overcome emerging resistance mechanisms while maintaining efficacy and tolerability. A summary of the key features of third-, fourth-, and allosteric EGFR inhibitors is provided in Table 1.

### 5.2. Combination Targeted Therapies

a. EGFR + MET Inhibition

Resistance to EGFR-TKIs often arises through bypass signaling, with MET amplification being a well-described driver of resistance that activates downstream pathways independent of EGFR [108]. To address this, dual inhibition of EGFR and MET has been investigated.

The INSIGHT trial evaluated the combination of tepotinib (a MET inhibitor) and gefitinib (an EGFR-TKI) in patients with EGFR-mutant NSCLC harboring MET amplification. The final analysis demonstrated that this combination resulted in improved PFS and overall survival (OS) compared to chemotherapy in this subgroup of patients. Notably, the combination was well-tolerated, with manageable adverse events, suggesting a favorable safety profile for this therapeutic strategy [109]. Similarly, a real-world study by Wu et al. reported meaningful activity of EGFR-TKI plus MET-TKI combinations (capmatinib or tepotinib) in 27 patients, yielding a 29.6% partial response rate, a median PFS of 7.3 months, and an OS of 26.9 months, with largely mild adverse events [110].

Hu et al. conducted a meta-analysis of 562 patients across six studies evaluating EGFR-TKI plus MET-TKI therapy in NSCLC with acquired MET alterations. The pooled results showed an objective response rate of 49.2%, disease control rate (DCR) of 78.6%, and median PFS of 5.6 months. Notably, combinations with third-generation EGFR-TKIs (e.g., osimertinib) outperformed first-generation agents in T790M-negative MET-driven resistance. Safety profiles were acceptable, with capmatinib-based regimens associated with lower hepatotoxicity and fewer grade ≥ 3 adverse events compared with savolitinib or tepotinib [111].

b. EGFR + HER2 Blockade

Another important mechanism of acquired resistance to EGFR-TKIs involves activation of the HER2 pathway, either through mutations or gene amplification, which can maintain proliferative signaling despite effective EGFR inhibition [112]. To overcome this bypass, dual targeting strategies combining EGFR-TKIs with HER2-directed therapies, including trastuzumab, or newer HER2 TKIs such as pyrotinib have been investigated, and early-phase clinical studies have demonstrated measurable antitumor activity.

For instance, a study by Gan et al. reported that in NSCLC patients with concomitant EGFR mutations and HER2 amplification, treatment with EGFR-TKI plus pyrotinib led to partial responses and disease stabilization, with a PFS ranging from 3 to 8 months [113].

Similarly, the TRAEMOS trial evaluated osimertinib combined with trastuzumab-emtansine (T-DM1) in patients with EGFR-mutant NSCLC exhibiting HER2 overexpression after progression on osimertinib. Although the overall objective response was limited, the combination demonstrated a manageable safety profile [114].

More recently, dual inhibition with almonertinib and pyrotinib has been reported to achieve prolonged survival in patients harboring EGFR mutations and HER2 amplification, while case reports of osimertinib combined with trastuzumab deruxtecan (T-DXd) have shown encouraging antitumor activity in the post-osimertinib setting [115,116].

c. EGFR + Anti-EGFR Antibodies

Combinations of EGFR-TKIs with anti-EGFR monoclonal antibodies such as cetuximab have been investigated as a strategy to enhance blockade of the EGFR signaling axis [117]. Cetuximab binds to the extracellular domain of EGFR, thereby preventing ligand-induced activation and receptor dimerization, while EGFR-TKIs inhibit the intracellular tyrosine kinase activity [118].

Evidence for this approach includes a Phase Ib study by Horn et al. in 2017, in which patients with progression on first- or second-generation EGFR-TKIs received afatinib followed by the addition of cetuximab [119]. The combination achieved a median PFS of 2.9 months and a median duration of response of 5.7 months. In this study, patients who had been on afatinib monotherapy for at least 12 weeks prior to combination therapy experienced improved outcomes, with a median PFS of 4.9 months [119]. Similar Phase 1b studies reported stable disease in 75% of patients and a median PFS of 2.7 months, confirming potential activity in diverse subtypes [120].

Randomized trials such as SWOG S1403 and ACE-Lung evaluated afatinib plus cetuximab versus afatinib alone in patients with common EGFR mutations (exon 19 deletions or L858R). Neither study demonstrated significant improvements in PFS or OS, indicating that dual EGFR blockade may not provide additional benefit for treatment-naïve patients [121,122]. Notably, in patients with EGFR exon 20 insertion mutations, the combination demonstrated more promising activity. A Phase II single-arm study reported a 54% DCR at 18 weeks, an overall response rate (ORR) of 43%, a median PFS of 5.5 months, and a median OS of 16.8 months [123].

Expanding beyond traditional EGFR-TKI resistance mechanisms, the addition of an ALK inhibitor has been explored in patients harboring EGFR cis-C797S mutations. This approach is based on preclinical evidence that dual EGFR/ALK targeting may overcome signaling bypasses induced by this specific resistance mutation. In a small retrospective cohort, the combination of cetuximab and brigatinib achieved a median PFS of 14 months, compared with 3 months for patients treated with standard chemotherapy, suggesting efficacy for dual EGFR/ALK inhibition in select cases [124].

Taken together, these studies highlight that afatinib plus cetuximab has a role in overcoming acquired resistance in some patients with NSCLC. While randomized first-line trials did not show benefit over monotherapy, this combination remains a guideline-supported option for EGFR-TKI-resistant NSCLC [125].

### 5.3. Targeting Downstream Signaling Pathways

a. PI3K/AKT/mTOR pathway inhibition

The PI3K/AKT/mTOR pathway is an important downstream effector of EGFR, promoting cell survival and proliferation [126]. Several inhibitors targeting this axis have been explored in combination with EGFR-TKIs.

PI3K inhibitors such as buparlisib, copanlisib, duvelisib, and pictilisib, as well as dual PI3K/mTOR inhibitors like dactolisib and apitolisib, have shown modest activity but were limited by dosing challenges and toxicity [126,127,128]. AKT inhibition with MK-2206 produced early evidence of antitumor activity in combination with EGFR-TKIs, while mTOR inhibitors such as everolimus demonstrated synergistic effects preclinically but only minimal clinical benefit with added toxicity in phase II trials [129,130,131].

Despite these encouraging laboratory results, clinical outcomes have been modest. In a multicenter phase II trial, patients receiving everolimus plus erlotinib demonstrated only minimal improvement in disease control and progression-free survival compared with erlotinib alone, while experiencing substantially higher rates of severe toxicities [132].

b. MAPK pathway targeting

The MAPK pathway is a another major downstream signaling cascade implicated in resistance to EGFR-TKIs in NSCLC. MEK1 and MEK2 serve as “gatekeeper” kinases that phosphorylate ERK1/2, regulating cell proliferation, differentiation, and apoptosis [133].

MEK inhibitors, such as trametinib, selumetinib, cobimetinib, and binimetinib, have been developed to target this pathway. While early-phase trials of MEK inhibitors as monotherapy in NSCLC showed limited efficacy and notable toxicities, combination strategies have demonstrated more promise [133].

In NSCLC patients with BRAF V600E mutations, combining the BRAF inhibitor encorafenib with binimetinib achieved an overall response rate of 75% in treatment-naïve patients, markedly improving outcomes compared to single-agent therapy, with an acceptable safety profile, leading to recent FDA approval for BRAF V600E–mutant metastatic NSCLC [133,134].

In ALK-positive NSCLC, studies demonstrated that adding MEK inhibitors such as trametinib or selumetinib to ALK inhibitors (e.g., crizotinib, ceritinib) enhances apoptosis and reduces proliferation, while minimizing toxicity when used at submaximal doses [133,134,135].

### 5.4. Immunotherapy Approaches

Combining EGFR-TKIs with immune checkpoint inhibitors (ICIs) has been explored as a strategy to overcome resistance in NSCLC, particularly in patients with high PD-L1 expression or tumor mutational burden following EGFR-TKI progression [136]. Preclinical studies suggest that EGFR inhibition can enhance tumor antigen presentation and T-cell infiltration, providing a rationale for combination therapy [137].

However, early clinical trials revealed significant safety concerns, most notably immune-related adverse events such as pneumonitis, hepatitis, and dermatologic toxicities, which have limited concurrent administration [138]. For instance, the TATTON trial (durvalumab + osimertinib) reported a 48% rate of grade ≥ 3 TRAEs and multiple cases of interstitial lung disease, leading to early termination of the study, while the CAURAL trial was also stopped prematurely after similar safety signals [139,140]. In CheckMate012, nivolumab plus erlotinib demonstrated modest efficacy (ORR 15%, 24-week PFS rate 48%) but was accompanied by toxicity-related withdrawals [95]. Likewise, combinations of pembrolizumab with first-generation TKIs (erlotinib or gefitinib) in KEYNOTE-021 showed variable efficacy but limited tolerability [141].

These results highlight the challenge of concurrent administration, as the enhanced immune activation induced by ICIs may exacerbate EGFR-TKI–related toxicities such as interstitial pneumonitis. Ongoing studies are evaluating optimal sequencing, dosing, and patient selection to maximize efficacy and safety [142].

### 5.5. Novel Modalities

a. Antibody–drug conjugates (ADCs)

ADCs are an emerging therapeutic class that link a monoclonal antibody specific for a tumor-associated antigen with a cytotoxic drug, thereby enabling targeted delivery of chemotherapy [143].

Patritumab deruxtecan (HER3-DXd) generated early enthusiasm in EGFR-mutant NSCLC. HER3 is broadly expressed in EGFR-mutant tumors and is implicated in tumor progression and resistance mechanisms. By delivering a potent topoisomerase I inhibitor payload directly to HER3-positive cells, HER3-DXd showed encouraging efficacy even in heavily pretreated patients [144].

In the phase II HERTHENA-Lung01 trial, HER3-DXd demonstrated clinically meaningful responses, including in patients who had progressed after osimertinib and chemotherapy, with a manageable safety profile [145]. However, in the subsequent phase III HERTHENA-Lung02 trial (NCT05338970), HER3-DXd did not meet its primary endpoint, and the manufacturer subsequently withdrew its FDA request for accelerated approval in EGFR-mutant NSCLC [146].

b. Bispecific antibodies

Bispecific antibodies are engineered to target two different receptors simultaneously, thereby enhancing antitumor activity and addressing bypass resistance mechanisms [147].

Amivantamab is a fully human bispecific antibody that binds both EGFR and MET, two key pathways involved in oncogenesis and drug resistance. It has already received FDA approval for the treatment of NSCLC with EGFR exon 20 insertion mutations based on the CHRYSALIS trial, where it demonstrated durable responses [73,148]. More recently, the role of amivantamab has expanded in the post-osimertinib setting for patients with common EGFR mutations. The MARIPOSA-2 trial established chemotherapy plus amivantamab as an FDA- and guideline-approved option after progression on osimertinib [149].

Combination approaches, such as amivantamab plus lazertinib (a third-generation EGFR TKI), have shown promising activity and are being evaluated in multiple clinical trials (e.g., MARIPOSA and CHRYSALIS-2). These studies may broaden the role of bispecific antibodies beyond exon 20 insertions to encompass acquired resistance following standard EGFR TKI therapy [150,151].

Together, these novel modalities represent a new frontier in the treatment of EGFR-mutant NSCLC, offering targeted strategies to overcome acquired resistance and expand therapeutic options beyond traditional EGFR-TKIs. A summary of ongoing studies evaluating these combination approaches is provided in Table 2. 

## 6. Conclusions

NSCLCs are heterogeneous tumors that are composed of numerous subclones. The analysis of these clones and their evolutionary trajectory during treatment reveals the complexity of resistance mechanisms, emphasizing that a comprehensive understanding of the molecular pathways involved is crucial for developing effective treatment strategies. Combining targeted therapies against multiple pathways (e.g., MET inhibitors in addition to EGFR inhibitors) or exploring novel approaches such as serial tumor sampling or liquid biopsies may be necessary to address resistance in these cases.

## Figures and Tables

**Table 1:** Overview of next-generation EGFR-targeted therapies.

Generation/Type	Example(s)	Target Mutations	Key Highlights
Third-Generation TKI	Osimertinib	T790M	Improved PFS and CNS efficacy; favorable safety profile
Fourth-Generation TKI	BLU-945, BLU-701	Includes C797S (esp. triple mutants)	Potent preclinical activity; some trials terminated (SYMPHONY—sponsor reasons; HARMONY—lack of efficacy)
Allosteric Inhibitors	EAI045, JBJ-04-125-02	T790M, C797S (non-ATP site binding)	EAI045: first-in-class, synergy with cetuximab but limited potency; JBJ-04-125-02: improved activity, effective against triple mutants; both remain preclinical

**Table 2:** Examples of ongoing clinical trials of combination and novel targeted therapies in EGFR-mutant NSCLC.

Combination	Trial Name	Phase	Status	Key Findings/Purpose
Amivantamab + Lazertinib	NCT06667076 (COPERNICUS)	Phase 2b	Recruiting	Evaluating the antitumor activity of amivantamab, a bispecific antibody targeting EGFR and MET, in combination with lazertinib, a third-generation EGFR-TKI, or with platinum-based chemotherapy in patients with EGFR-mutant locally advanced or metastatic NSCLC.
Amivantamab + Lazertinib or Chemotherapy	NCT02609776 (CHRYSALIS)	Phase 1/2	Active, not recruiting	Evaluating the safety, pharmacokinetics, and preliminary efficacy of amivantamab alone and in combination with lazertinib or chemotherapy in patients with advanced NSCLC
MCLA-129 (Anti-EGFR/c-MET Bispecific) + EGFR-TKI	NCT04868877	Phase 1/2	Recruiting	Investigating the combination of MCLA-129, a bispecific antibody targeting EGFR and c-MET, with EGFR-TKIs in advanced NSCLC and other solid tumors.
Inetetamab + Pyrotinib	NCT05016544	Phase 1	Unknown status	Evaluating the safety and efficacy of inetetamab, an anti-EGFR monoclonal antibody, combined with pyrotinib, a HER2-targeted therapy, in patients with HER2-mutant or amplified NSCLC.
Amivantamab + Lazertinib	NCT04077463 (CHRYSALIS-2)	Phase 1/2	Active, not recruiting	Confirming the tolerability of lazertinib and evaluating its combination with amivantamab in patients with advanced NSCLC.
ELVN-002 (Anti-HER2 ADC)	NCT05650879	Phase 1a/1b	Active, not recruiting	Testing ELVN-002, an anti-HER2 antibody-drug conjugate, in patients with HER2-altered cancers, including NSCLC.
Savolitinib + Osimertinib	NCT05261399 (SAFFRON)	Phase 3	Recruiting	Comparing savolitinib (a MET-TKI) + osimertinib versus platinum-based chemotherapy in EGFR-mutant, MET-altered NSCLC after progression on osimertinib.
Zongertinib (HER2-TKI)	NCT04886804 (Beamion LUNG-1)	Phase 1	Recruiting	Dose-escalation study in HER2-altered tumors; part 2 tests antitumor activity in HER2-mutant NSCLC.
Necitumumab + Osimertinib	NCT02496663	Phase 1	Active, not recruiting	Studied the combination of necitumumab, an anti-EGFR monoclonal antibody, with osimertinib in patients with EGFR-mutant NSCLC.

Abbreviations: NSCLC—non-small cell lung cancer/TKI—tyrosine kinase inhibitor/ADC—antibody–drug conjugate/MET—mesenchymal–epithelial transition factor/HER2—human epidermal growth factor receptor/EGFR—epidermal growth factor receptor. Table compiled from references [52,152,153,154,155,156,157,158].

## Data Availability

No new data were created or analyzed in this study. Data sharing is not applicable to this article.
